# mDIXON-Quant for differentiation of renal damage degree in patients with chronic kidney disease

**DOI:** 10.3389/fendo.2023.1187042

**Published:** 2023-07-21

**Authors:** Yue Wang, Ye Ju, Qi An, Liangjie Lin, Ai Lian Liu

**Affiliations:** ^1^ First Affiliated Hospital, Dalian Medical University, Dalian, China; ^2^ Clinical and Technical Support, Philips Healthcare, Beijing, China

**Keywords:** MRI, mDIXON-Quant, R2*, fat fraction, chronic kidney disease

## Abstract

**Background:**

Chronic kidney disease (CKD) is a complex syndrome with high morbidity and slow progression. Early stages of CKD are asymptomatic and lack of awareness at this stage allows CKD to progress through to advanced stages. Early detection of CKD is critical for the early intervention and prognosis improvement.

**Purpose:**

To assess the capability of mDIXON-Quant imaging to detect early CKD and evaluate the degree of renal damage in patients with CKD.

**Study type:**

Retrospective.

**Population:**

35 patients with CKD: 18 cases were classifified as the mild renal damage group (group A) and 17 cases were classifified as the moderate to severe renal damage group (group B). 22 healthy volunteers (group C).

**Field strength/sequence:**

A 3.0 T/T_1_WI, T_2_WI and mDIXON-Quant sequences.

**Assessment:**

Transverse relaxation rate (R2*) values and fat fraction (FF) values derived from the mDIXON-Quant were calculated and compared among the three groups.

**Statistical tests:**

The intra-class correlation (ICC) test; Chi-square test or Fisher’s exact test; Shapiro-Wilk test; Kruskal Wallis test with adjustments for multiplicity (Bonferroni test); Area under the receiver operating characteristic (ROC) curve (AUC). The significance threshold was set at P < 0.05.

**Results:**

Cortex FF values and cortex R2* values were significantly different among the three groups (P=0.028, <0.001), while medulla R2* values and medulla FF values were not (P=0.110, 0.139). Cortex FF values of group B was significantly higher than that of group A (Bonferroni adjusted P = 0.027). Cortex R2* values of group A and group B were both significantly higher than that of group C (Bonferroni adjusted P = 0.012, 0.001). The AUC of cortex FF values in distinguishing group A and group B was 0.766. The diagnostic efficiency of cortex R2* values in distinguishing group A vs. group C and group B vs. group C were 0.788 and 0.829.

**Conclusion:**

The mDIXON-Quant imaging had a potential clinical value in early diagnosis of CKD and assessing the degree of renal damage in CKD patients.

## Highlights

1. mDIXON-Quant imaging may provide a reference for the early intervention and diagnosis of CKD.

2. The R2* and FF values of mDIXON-Quant imaging can reflect the degree of tissue hypoxia and lipid deposition in CKD with different degrees of renal damage;

3. The R2* values have good sensitivity and specificity in the early diagnosis of CKD.

## Introduction

Chronic kidney disease (CKD) is a complex syndrome with a high morbidity (affects approximately 10-13% of the world’s population) (1). It is characterized by the irreversible changes in renal function and structure, and slow progression of the disease (2). According to the Kidney Outcomes Quality Initiative (KDIGO) CKD guideline (1, 3), CKD is defined as glomerular filtration rate (GFR) <60 mL/min/1.73 m^2^ lasting at least 3 months, or GFR≥60 mL/min/1.73 m^2^, but with evidence of injury of the renal structure. Early stages of CKD are asymptomatic and patients have no clinical manifestation in most cases (1, 4). Lack of awareness and intervention at this stage allows CKD to progress through to advanced stages of the disease, even may reach an endpoint of end-stage kidney disease (ESKD) or uremia (4) requiring renal replacement therapy, as well as causing a significant clinical and economic burden. Therefore, the early detection of CKD renal function is critical for the timely treatment of the disease, and is helpful for improving outcomes and decreasing mortality.

In clinical routine practice, renal function has been commonly monitored by the GFR based on blood serum creatinine level (sCr) or by the gold standard kidney biopsy. However, those methods have some limitations and drawbacks. GFR lacks sensibility and accuracy, and cannot offer a split renal function measurement. Kidney biopsy is invasive and have a risk of bleeding and pain. Besides, the sampling limitations may lead to bias of the diagnosis (5, 6).

Functional magnetic resonance imaging (fMRI) provides comprehensive tools for noninvasive evaluation of renal function including diffusion, perfusion, oxygenation, hemodynamics and others (6, 7). In CKD, chronic hypoxia and lipid metabolism abnormality have been recognized to play a pivotal role. Peritubular capillaries injury, constant oxygen consumption and inflammation induce low renal perfusion and hypoxia damage (8). Deregulated fatty acid metabolism and renal lipid accumulation cause inflammation, oxidative stress and fibrosis, exacerbating existing kidney damage (9). Blood oxygen level-dependent (BOLD) MRI is a noninvasive imaging method to assess oxygenation changes and has been widely used in different kidney diseases (10–12). It is based on the paramagnetic deoxyhemoglobin. An increase in the R2* value indicates a decrease of local tissue oxygen content. However, it cannot assess kidney fat deposition quantitatively.

mDIXON-Quant is a non-invasive three-dimensional (3D) multi-echo gradient-echo (GRE) sequence, generating water, fat, water-fat in-phase, water-fat anti-phase as well as the transverse relaxation rate (R2*) and fat fraction (FF) images (13). R2* is proportional to the deoxyhemoglobin content, which can indirectly reflect the partial pressure of oxygen in the local tissue. FF value can assess fat content quantitatively (14). mDIXON-Quant has been applied in the fat quantitative research of the liver (15, 16) and skeletal system (17, 18), etc. Only a limited number of studies concern the application of renal fat quantitative imaging in CKD (19, 20). To our knowledge, the studies of using mDIXON-Quant imaging to evaluate the degree of renal function impairment is extremely lacking.

Hence, the aim of the study was to assess the capability of mDIXON-Quant imaging to detect early CKD and evaluate the degree of renal damage in patients with CKD.

## Materials and methods

### Subjects

Patients with chronic kidney disease who underwent 3.0 T MR scans from August 2019 to October 2020 were retrospectively collected. Inclusion criteria were: (1) age >18 years; (2) CKD, in line with the definition of kidney disease proposed by the KDIGO (3), and the aetiology included type II diabetes, hypertension, IgA nephropathy, chronic glomerulonephritis etc.; (3) received complete MR scans, including conventional renal MR scan sequences (T_1_WI, T_2_WI) and mDIXON-Quant sequence. Exclusion criteria were: (1) kidney stones, hydronephrosis, renal tumor, polycystic kidney disease, and other renal occupational diseases; (2) taking drugs that may affect creatinine levels (such as cimetidine, trimethoprim, or cefotaxime) or receiving renal replacement therapy; (3) poor image quality:the images were not clear enough or had respiratory and motion artifacts; (4) severe metabolic syndrome, insulin resistance, etc., or receiving related treatments that cause nutritional, metabolic disorders such as parenteral nutrition, taking glucocorticoids, etc. (5) body mass index (BMI) ≥ 30 kg/m^2^ (the people who were considered obese) (21), or use of other drugs which may affect the lipid metabolism such as statin therapy. Finally, 35 CKD patients (Asian, 18 males and 17 females) were enrolled.

At the same time, 22 healthy volunteers were included in the control group (group C: Asian, 5 males and 17 females, average age 33.74 ± 11.63 years old, range 24-60 years old). Inclusion criteria were the following: (1) healthy adults over 18 years old who had undergone regular physical examinations; (2) had no previous history of urinary system diseases, systemic metabolic or endocrine diseases, diabetes, or hypertension; (3) had no contraindications of MRI examination. Exclusion criteria were: (1) renal insufficiency caused by renal space-occupying lesions, hydronephrosis, and infectious lesions confirmed by MRI; (2) taking vascular or nephrotoxic drugs within the first three months of examination; (3) poor image quality:the images were not clear enough or had respiratory and motion artifacts.

**Figure 1:**
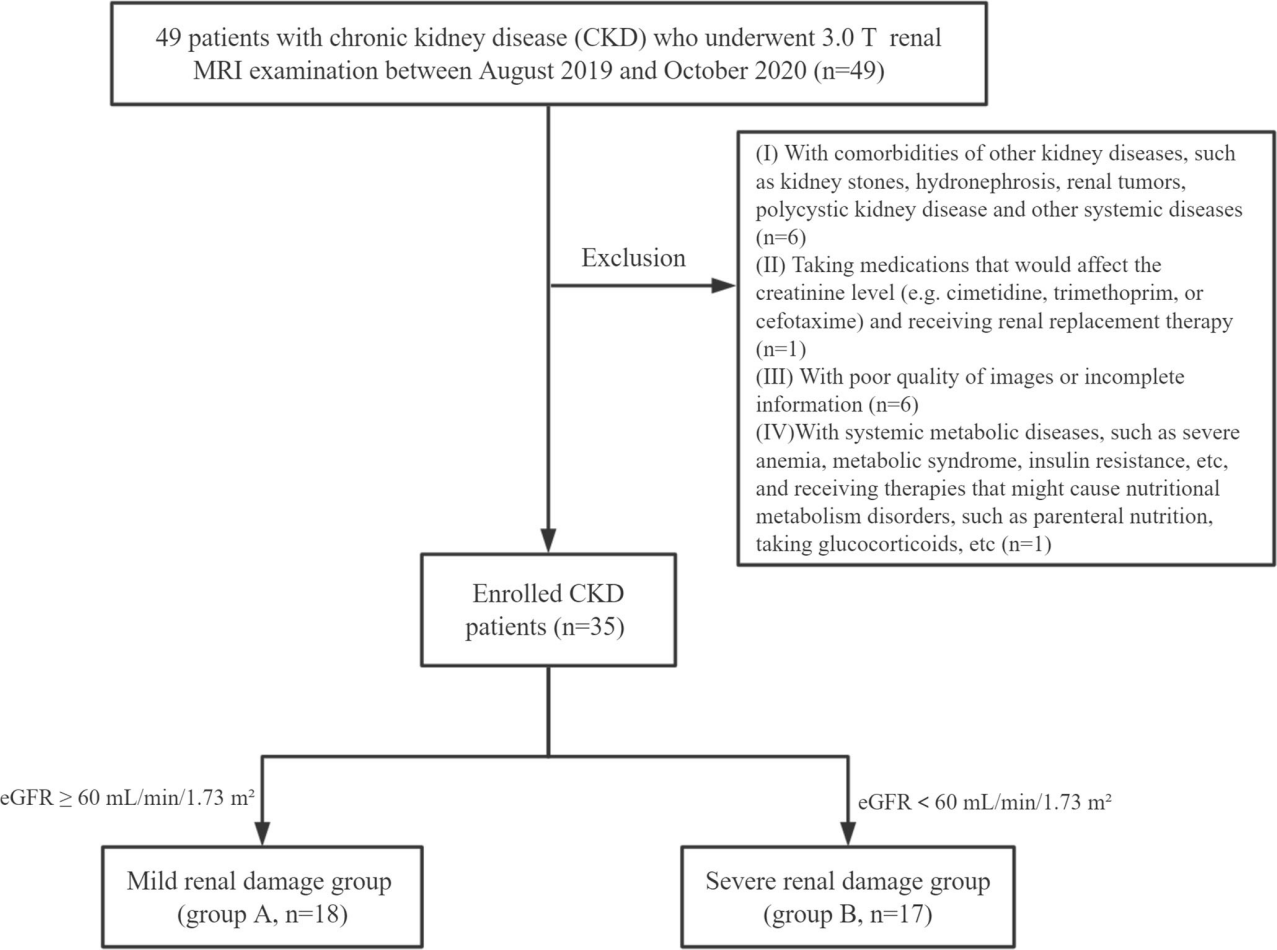
Flow chart of study population in the study.

Finally, all the participants were Asian people. The flow chart of study population in the study is shown in **Figure 1**.

### MR scanning protocol

Kidney MR scans were performed with a 3.0 T MR scanner (Ingenia CX, Philips Healthcare, Best, the Netherlands) using a 32-channel abdominal coil. All subjects were required to fast for more than 6 hours before scanning. The mDIXON-Quant sequence acquired under multiple breath-holds, and the flip angle was 3 degree. The scan parameters of T1WI, T2WI and mDIXON-Quant sequence were shown in **Table 1**.

**Table 1:** Scan parameters.

Sequences	Scan Duration (s)	Flip Angle	TR/TE (ms)	FOV(mm)	ACQ Voxel Size (mm)	Slice Thickness/ Slice gap (mm)
T_1_WI	90	15°	10/2	392	1.40/1.95/6.00	6.0/-2.0
T_2_WI	150	90°	1500/117	380	0.88/0.88/6.00	6.0/1.0
mDIXON-Quant	15	3°	6/1.04	375	2.29/1.85/5.00	5.0/-2.5

T_1_WI, T_1_ weighted imaging; T_2_WI, T_2_ weighted imaging; TR, time of repetition; TE, time of echo; FOV, field of view; ACQ, actual acquisition.

### Image analysis and data measurement

All images were transferred to the ISP (Intellispace Portal 9, Philips Healthcare) workstation for analysis. The post-processing of mDixon-Quant imaging was performed on the MR console after data collection. After phase correction, accurate fat quantification was achieved with a seven-peak spectral fat model that enabled T2* corrections (22). The proton density fat fraction (FF) map was computed as the ratio of the fat signal over the sum of fat and water signals. We performed an mDIXON-Quant image analysis on the right kidney. The reasons are: (1) the anatomical position of the right kidney is relatively fixed and is less affected by respiratory movement and intraperitoneal intestinal gas compared to the left kidney; (2) to ensure the homogeneity of the B0 and B1 fields. The evaluation was performed independently by two radiologists with 2 and 8 years experiences in abdominal imaging diagnosis. They were both blinded to the clinical and imaging information. The first step was to record whether the kidneys had cysts or other space occupying lesion. When there was a disagreement, the result of the negotiation was decided. Second, the two individuals completed the measurement of mDixon-Quant data independently. They respectively identified the renal cortex and medulla and other anatomical structures based on T_1_WI and T_2_WI, and selected a slice in the upper, middle, and lower poles of the right kidney. The six ROIs were carefully placed on the cortex and medulla, with an area of about 10-25 mm^2^, avoiding renal sinus, large blood vessels, and perirenal tissue. Finally, the averaged R2* and FF values from three levels of cortex or medulla were measured and analyzed.

### Assessment of GFR

Serum creatinine (Scr) values were measured on the day of the MRI examination for all subjects. GFR was calculated using the Modification of Diet in Renal Disease (MDRD) formula (23): GFR (ml/min/1.73 m²) = 175 × (Scr/88.4)^-1.154^ × (Age)^-0.203^ × (0.742 if female). Patients with CKD were divided into two subgroups according to GFR (1, 3): mild renal damage group (group A, GFR ≥ 60 ml/min/1.73 m^2^); moderate to severe renal damage group (group B, GFR<60 mL/min/1.73 m^2^).

### Statistical analysis

Data were analyzed by SPSS 26.0 (IBM, Armonk, NY, USA) and MedCalc 11.4 (MedCalc, Mariakerke, Belgium). We have performed repeated measurements of mDIXON-Quant parameters for two radiology doctors (with 8 years and 2 years of experiences in abdominal imaging) to analyze the inter- and intra-observer variability, and intra-class correlation (ICC) under a two-way random model with the absolute agreement was applied for the assessment. ICC values lower than 0.40, between 0.40 and 0.75 and greater than 0.75 were considered to have low, medium, and high consistency, respectively. Categorical variables presented as counts or percentages were compared using a chi-square test or Fisher’s exact test. The Shapiro-Wilk test was used to test the normality of the continuous variables. According to the distribution of the parameters, the paramters were expressed as mean ± standard deviation (normal distribution) or median with interquartile range (non-normal distribution). The parameters among three groups were analyzed using Kruskal Wallis test. *Post-hoc* multiple pairwise comparisons were performed with the Bonferroni test. Receiver operating characteristic (ROC) curve analysis was used to analyze the diagnostic efficacy of the parameters and their combination to evaluate the renal function.

This study was approved by the Medical Ethics Committee of our hospital (approval number: PJ-KS-KY-2021-250).

## Results

### Demographics and CKD stages of subjects

According to the GFRs, CKD patients were divided into mild renal damage group (group A, 18 cases, 9 males and 9 females, average age 46.33 ± 15.27 years old, range 19-75 years old), and moderate to severe renal damage group (group B, 17 cases, 9 males and 8 females, mean age 45.94 ± 14.89 years old, range 27-74 years old). The GFRs of healthy volunteers were all within normal limits.

CKD is categorized into five stages according to the GFR (1). The demographics of all subjects and CKD stages of the patients were shown in **Table 2**.

**Table 2:** The demographics of all subjects and CKD stages of the patients.

Class	Number	Age (range, mean± standard deviation)	GFR (ml/min/1.73 m²)
Healthy volunteer	22	24-60, 33.73 ± 11.63	/
CKD 1	9	19-60, 44.11 ± 14.56	120.91 ± 15.84
CKD 2	9	23-75, 48.56 ± 16.50	78.86 ± 6.89
CKD 3	2	34-59, 46.50 ± 17.68	42.82 ± 14.17
CKD 4	4	34-74, 56.50 ± 16.67	22.79 ± 5.157
CKD 5	11	27-70, 42.00 ± 13.36	8.03 ± 2.78

CKD, Chronic Kidney Disease; GFR, glomerular filtration rate; CKD 1, GFR ≥ 90 ml/min/1.73 m²; CKD 2, 60 ml/min/1.73 m² < GFR≤ 89 ml/min/1.73 m²; CKD 3, 30 ml/min/1.73 m² < GFR ≤ 59 ml/min/1.73 m²; CKD 4, 15 ml/min/1.73 m² < GFR ≤ 30 ml/min/1.73 m²; CKD 5, GFR < 15 ml/min/1.73 m².

### Renal T_2_WI, R2*, and FF images in healthy volunteers and CKD patients

**Figure 2:**
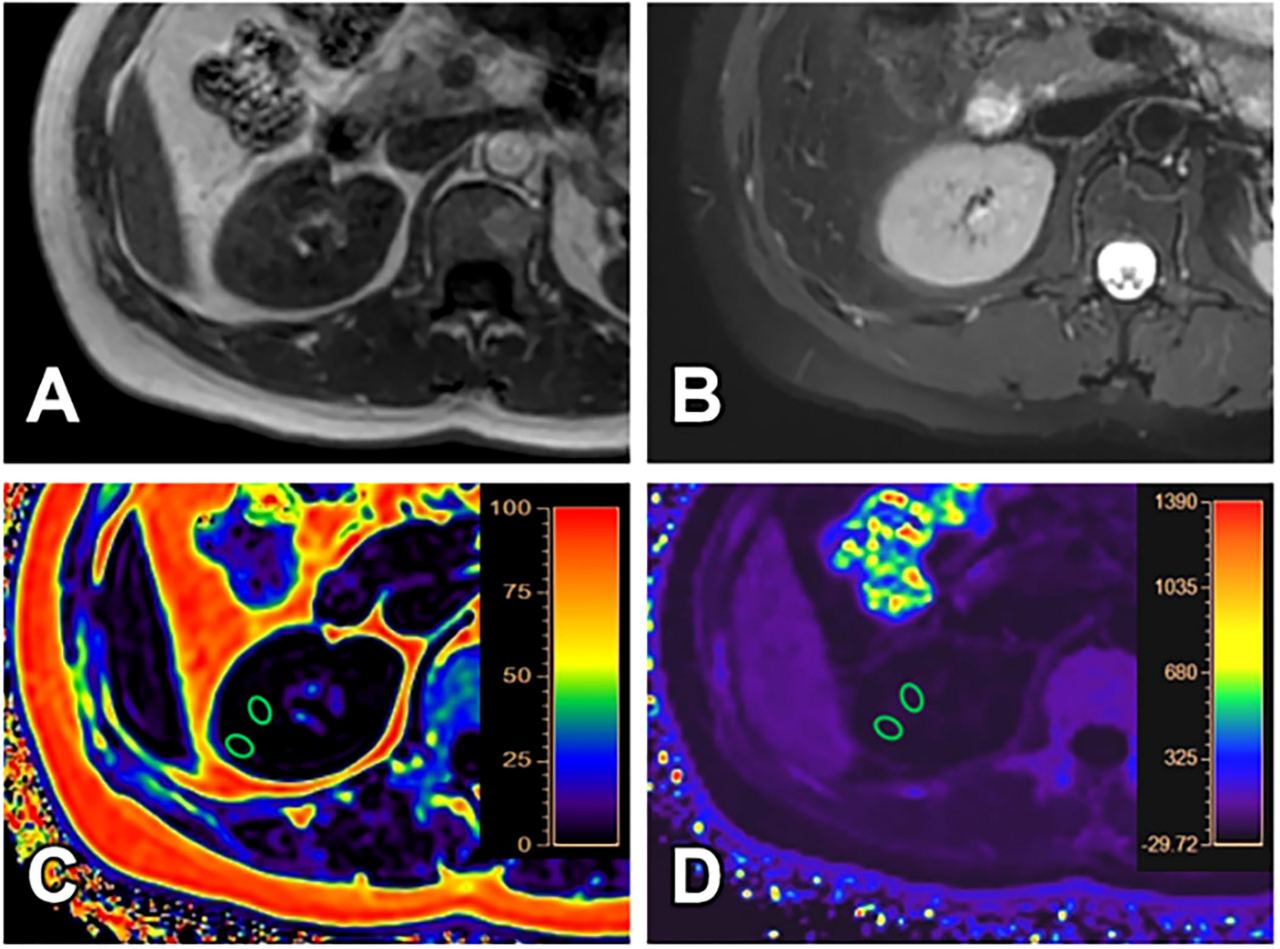
A 40-year-old female with GFR (128.11 ml/min/1.73 m^2^) and clinical CKD stage I (mild renal damage group). T_1_WI **(A)**, T_2_WI **(B)**, FF **(C)** and R2* **(D)** images. The cortex and medulla R2* values are 16.17/s and 21.52/s; the cortex and medulla FF values are 0.61% and 0.64%, respectively.

**Figure 3:**
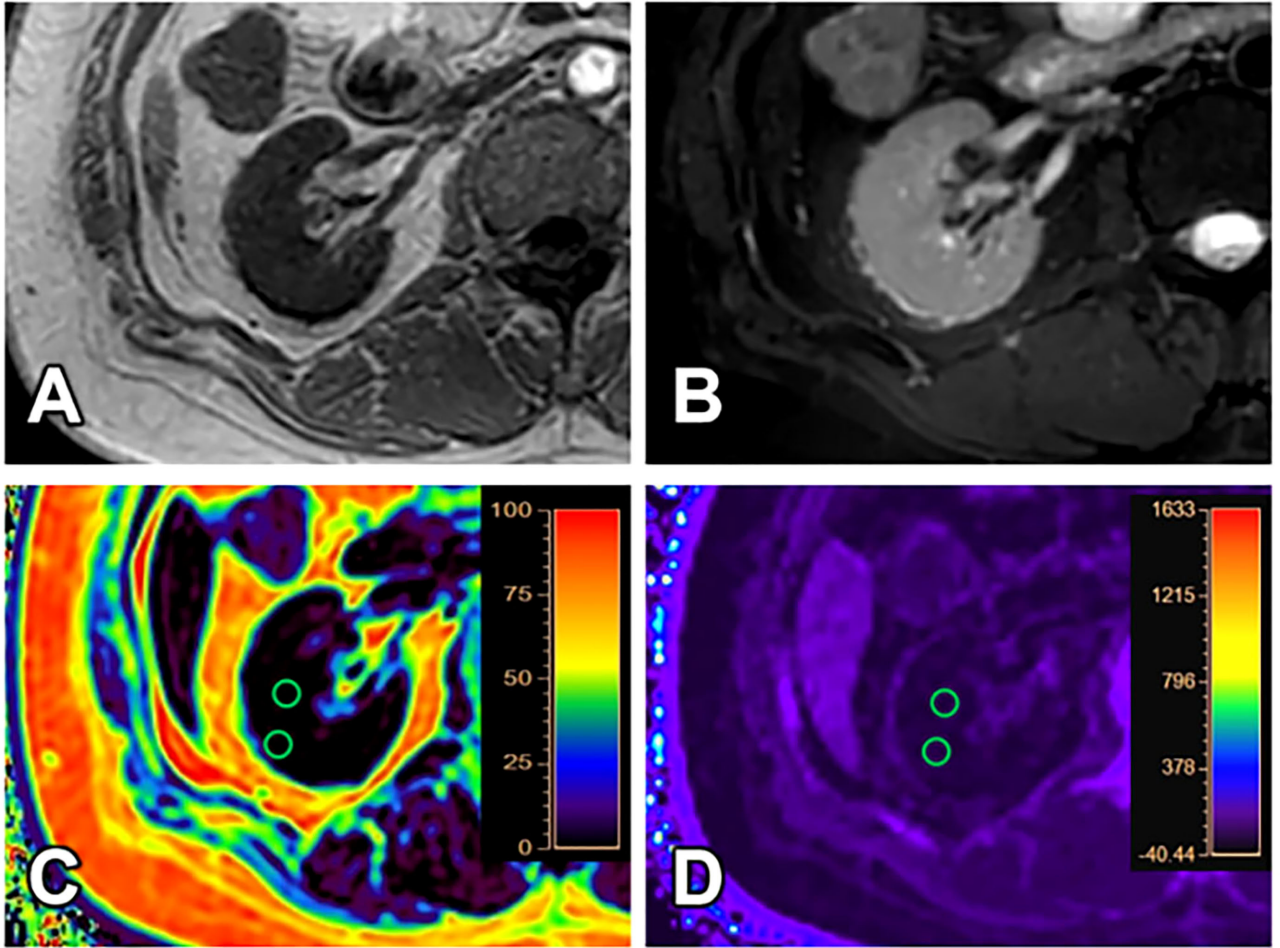
A 31-year-old female with GFR (8.16 ml/min/1.73 m2) and clinical CKD stage IV (moderate to severe renal damage group). T_1_WI **(A)**, T_2_WI **(B)**, FF **(C)** and R2* **(D)** images. The cortex and medulla R2* values are 18.87/s and 15.90/s; the cortex and medulla FF values are 1.30% and 1.79%, respectively.

**Figure 4:**
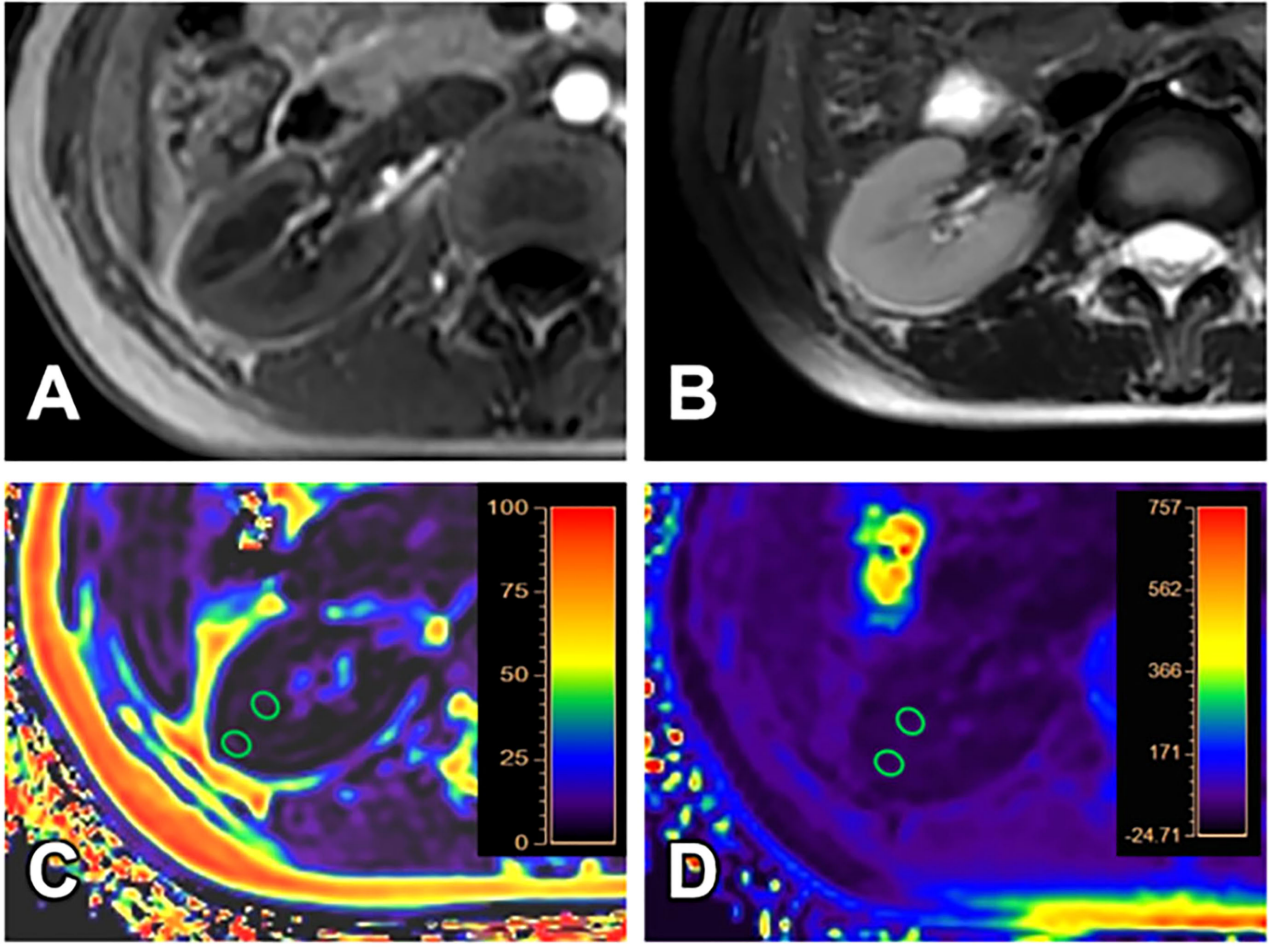
A 24-year-old female volunteer (healthy volunteer group). T_1_WI **(A)**, T_2_WI **(B)**, FF **(C)** and R2* **(D)** images. The cortex and medulla R2* values are 19.27/s and 21.67/s; the cortex and medulla FF values are 1.32% and 1.82%, respectively.

All images have adequate quality for structure visualization and data measurements. Representative kidney T_1_WI, T_2_WI, R2*, and FF maps in different groups were shown in **Figures 2**–**4**.

### Measurement consistency between two observers

The ICC values of each parameter between the two observers were all > 0.75, suggesting good consistency. The data measured by observer 1 (with longer years of experience) was then used for subsequent data analysis. The Shapiro-Wilk test results showed that the data was not in normal distribution and was expressed as median (25th percentile, 75th percentile). The parameters of the three groups and ICC tests results were shown in **Table 3**.

**Table 3:** Intra-observer agreement on the measurement of imaging parameters.

Parameters	Groups	Observer 1	Observer 2	ICC values
Cortex FF values (%)	Group A (n = 18)	1.13 (0.61, 1.58)	1.06 (0.70, 1.65)	0.915
	Group B (n = 17)	1.72 (1.31, 2.61)	1.60 (1.31, 2.49)	0.997
	Group C (n = 22)	1.19 (0.90, 2.01)	1.23 (0.79, 2.14)	0.987
medulla FF values (%)	Group A (n = 18)	0.90 (0.63, 1.73)	1.22 (0.90, 2.39)	0.854
	Group B (n = 17)	1.71 (0.87, 4.26)	2.56 (1.50, 3.78)	0.942
	Group C (n = 22)	1.28 (0.65, 2.02)	1.42 (1.07, 2.72)	0.821
cortex R2* values (/s)	Group A (n = 18)	17.23 (16.14, 19.02)	18.21 (16.37, 19.59)	0.763
	Group B (n = 17)	18.81 (16.55, 20.05)	19.31 (17.80, 20.05)	0.954
	Group C (n = 22)	15.38 (14.45, 16.92)	17.04 (14.69, 18.14)	0.857
medulla R2* values (/s)	Group A (n = 18)	21.60 (19.28, 23.53)	21.69 (17.48, 26.59)	0.860
	Group B (n = 17)	21.34 (17.60, 22.80)	21.56 (17.48, 23.62)	0.972
	Group C (n = 22)	19.82 (19.01, 20.73)	22.52 (19.46, 23.42)	0.759

Observer 1 and 2, two radiologists with 8 years and 2 years of experiences in abdominal imaging; ICC, intra-class correlation; ICC values lower than 0.40, between 0.40 and 0.75 and greater than 0.75 were considered to have low, medium, and high consistency, respectively; Group A, mild renal damage group; group B, moderate to severe renal damage group; group C, healthy control group; FF, fat fraction; R2*, transverse relaxation rate.

### Comparison of FF values and R2* values of the cortex and medulla among the three groups

The Kruskal Wallis test showed that cortex FF values and cortex R2* values were significantly different among the three groups (P=0.028, <0.001), while medulla R2* values and medulla FF values were not (P=0.110, 0.139).

**Figure 5:**
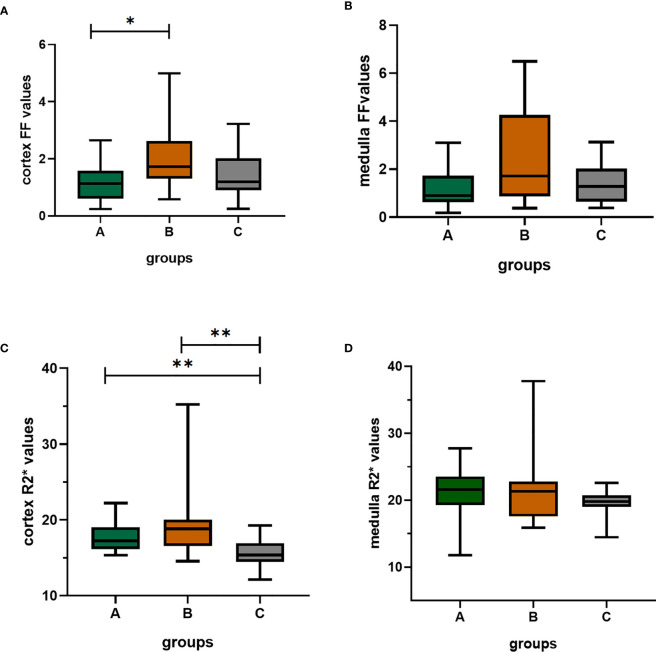
The box-plots of FF(%) values and R2*(/s) values of the cortex and medulla in the three groups. **(A–D)** showed the differences among three groups, respectively. There was significant difference in cortex FF values between group A and group B; the group A and group B both had significantly differences with group C; Bonferroni adjusted P values (adj. P): *, adj. P < 0.05; **, adj. P < 0.01. Group A, mild renal damage group; group B, moderate to severe renal damage group; group C, healthy control group; FF(%), fat fraction; R2*(/s), transverse relaxation rate.

The cortex FF values of the moderate to severe renal damage group (group B) was significantly higher than that of the mild renal damage group (group A) (Bonferroni adjusted P = 0.027). The cortex R2* values of group A and group B were both significantly higher than that of group C (Bonferroni adjusted P = 0.012, 0.001) (**Figure 5**).

### The diagnostic efficacy of the parameters in evaluating the renal function of CKD patients and healthy volunteers

The diagnostic efficiency of cortex FF values in distinguishing group A and group B was 0.766, with a sensitivity and specificity of 66.7% and 82.4%, respectively, which means that cortex FF values may be helpful to distinguish the degree of renal function damage in CKD patients and stage the diseases. Meanwhile, the diagnostic efficiency of cortex R2* values in distinguishing group A and group C was 0.788, with sensitivity and specificity of 88.9% and 68.2%, respectively, which reflects that cortex R2* values have the potential for early diagnosis of CKD. Besides, the diagnostic efficiency of cortex R2* values in distinguishing group B and group C was 0.829, with sensitivity and specificity of 70.6% and 81.8%, respectively, which means cortex R2* values can noninvasively distinguish patients with severe CKD from healthy volunteers.

**Figure 6:**
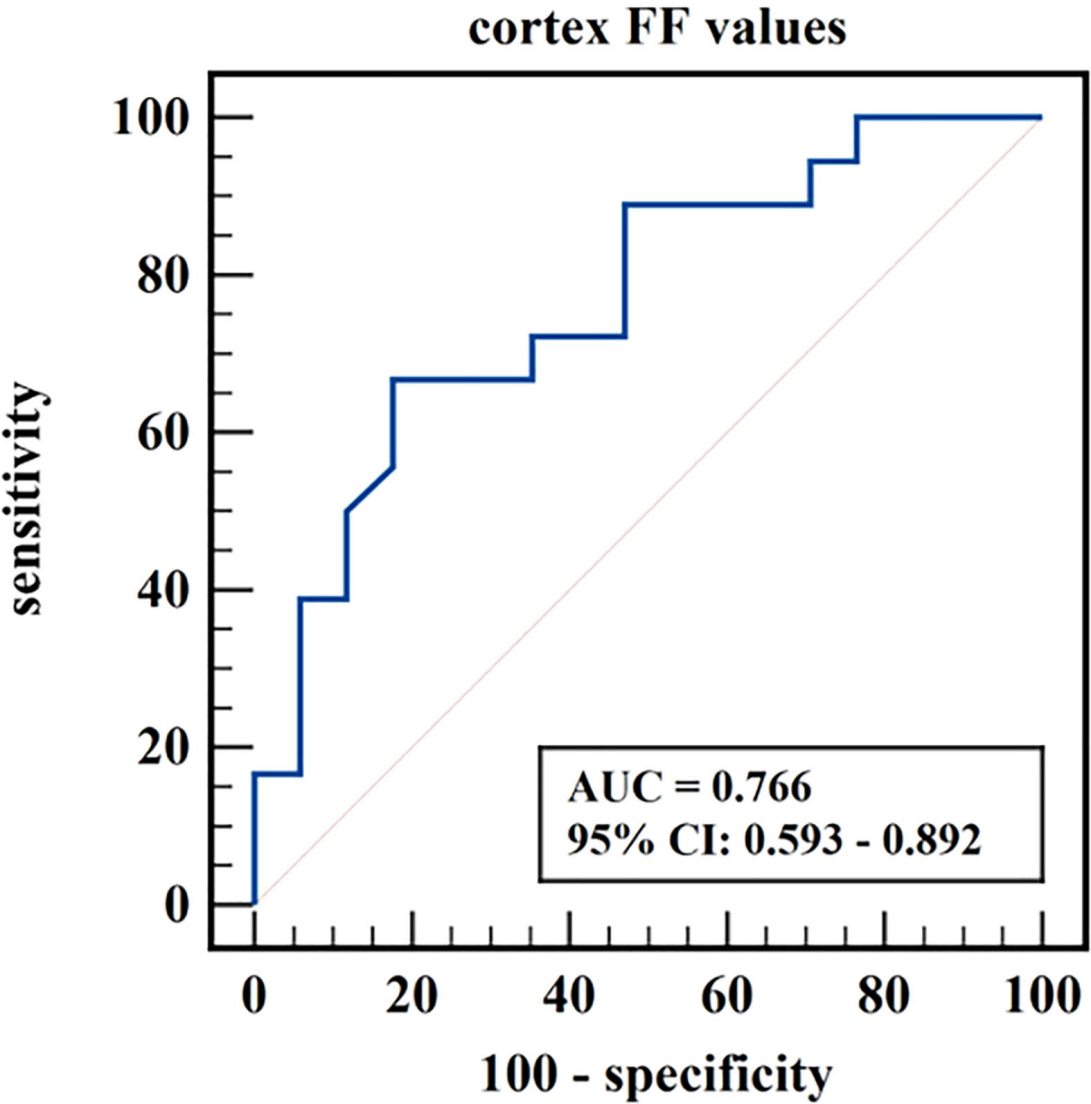
ROC curve of cortex FF values in distinguishing mild renal damage group from moderate to severe renal damage group.

**Figure 7:**
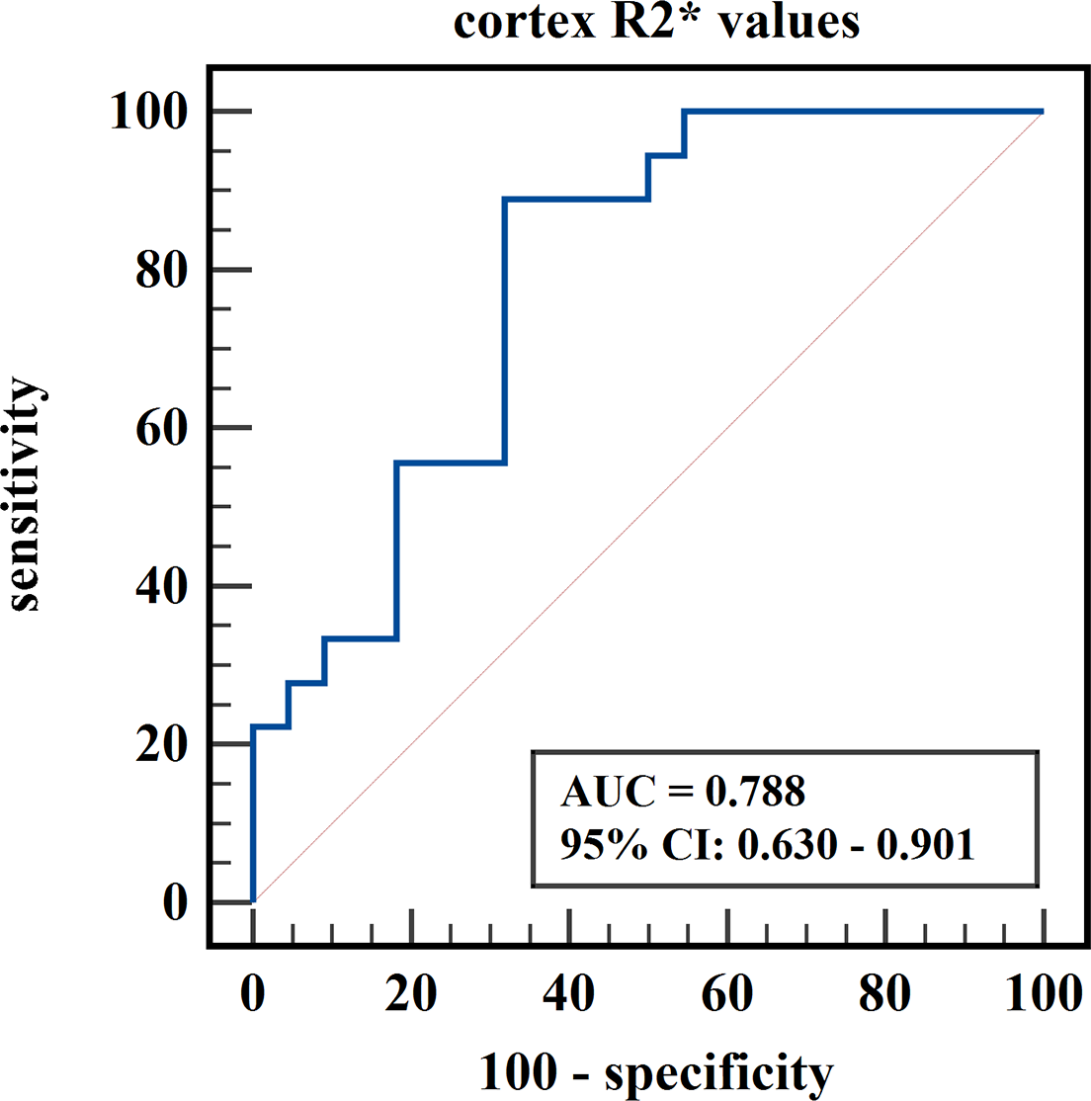
ROC curve of cortex R2* values in distinguishing mild renal damage group from healthy control group.

**Figure 8:**
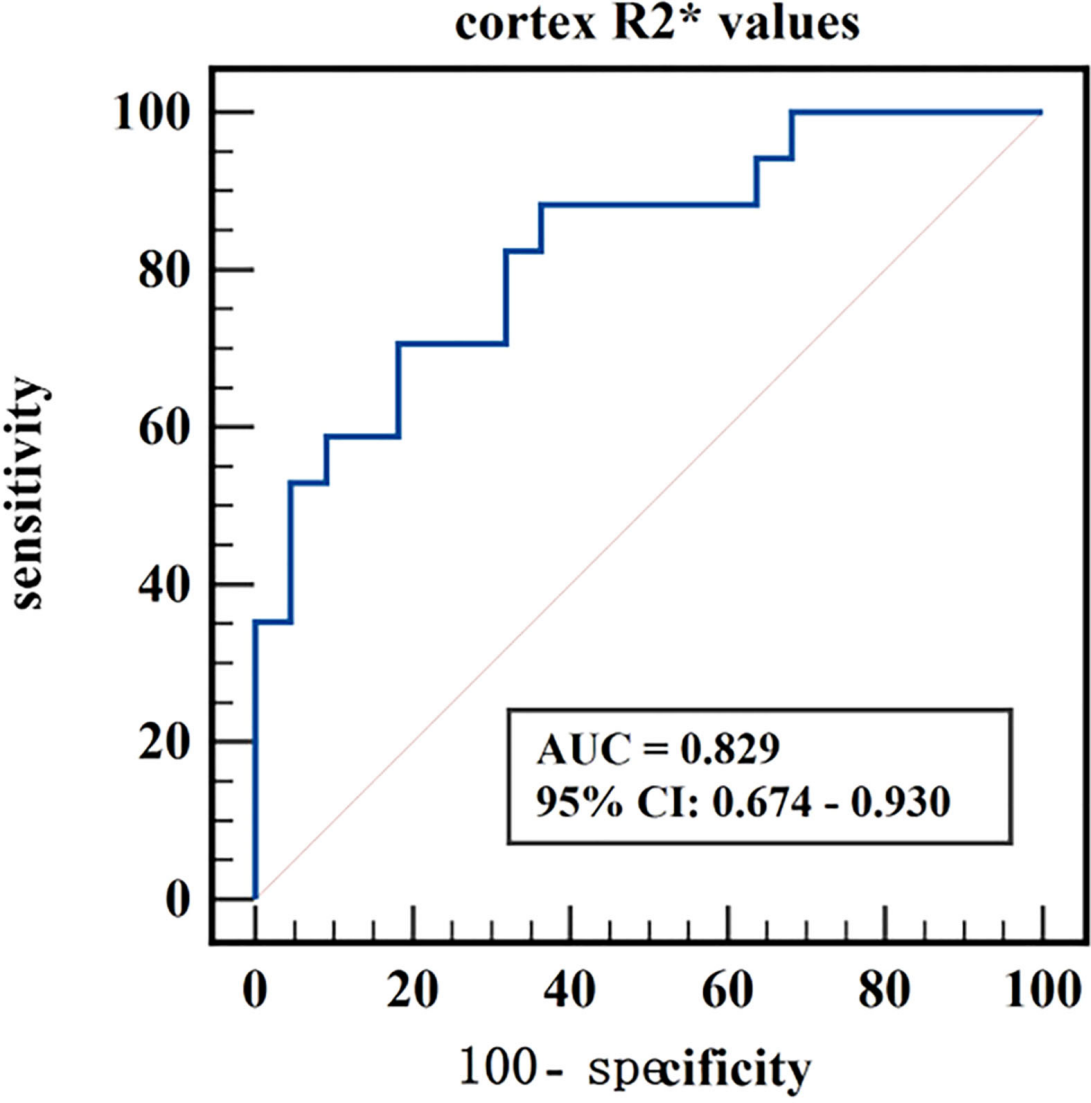
ROC curve of cortex R2* values in distinguishing moderate to severe renal damage group from healthy control group.

The AUC values, 95% confidence interval (CI), cutoff values, sensitivities, and specificities of the parameters in evaluating the renal function of CKD patients and healthy volunteers were shown in **Table 4**. The ROC curves were shown in **Figures 6**–**8**.

**Table 4:** Sensitivities, specificities, and area under curve (AUC) values of the parameters in evaluating the degree of renal damage in CKD.

Group vs. Group	Parameter	AUC	95% CI	Cutoff value	Sensitivity (%)	Specificity (%)
Group A vs. Group B	cortex FF (%)	0.766	0.593 - 0.892	1.27	66.7	82.4
Group A vs. Group C	cortex R2* (/s)	0.788	0.630 - 0.901	15.87	88.9	68.2
Group B vs. Group C	cortex R2* (/s)	0.829	0.674 - 0.930	16.97	70.6	81.8

Group A, mild renal damage group; group B, moderate to severe renal damage group; group C, healthy control group; CI, confidence interval; AUC: area under curve; FF, fat fraction.

## Discussion

We explored the renal R2* and FF values derived from mDIXON-Quant imaging in evaluating the renal function of CKD patients with different degrees of kidney damage and healthy volunteers. The results showed that the cortex R2* values of the mild and moderate to severe renal damage groups were both higher than that of the healthy control group; the cortex FF values in the moderate to severe renal damage group were significantly higher than in the mild renal damage group; there was no significant difference in medulla R2* values and medulla FF values among the three groups. The mDIXON-Quant imaging had a potential clinical value in assessing the degree of renal damage in CKD patients.

### The cortex R2* values of the mild and moderate to severe renal damage groups were higher than that of the healthy control group

The R2* values are proportional to the concentration of deoxyhemoglobin, which can indirectly reflect the partial pressure of oxygen in local tissues. This study showed that the cortex R2* values of the mild and moderate to severe renal damage groups were higher than that of the healthy control group, which was consistent with the results of previous related studies (24–27). The possible reasons are: CKD is accompanied by different degrees of glomerular atrophy and tubular fibrosis, which may change the local hemodynamics of the kidney and cause damage to the capillary endothelium and microvessels, resulting in decreased renal perfusion and chronic hypoxia. The renal cortex is rich in capillaries, and the renal blood flow reduction in CKD mainly occurs in the cortex (28); besides, the proximal tubular cells are the predominant cell type in the cortex. This kind of cells have a large number of mitochondria and have active transport activity which consumes a lot of energy and oxygen. So the cortex is more susceptible to the level of oxygenation and hypoxic injury, which is consistent with the cortex R2* values ​​in the mild and moderate to servere renal damage group being higher than those in the healthy control group, but the medulla R2*values have no significantly difference among the three groups. Therefore, the cortex R2* values reflect the degree of renal hypoxia and have the potential for noninvasively early diagnosis of CKD. It also can effectively distinguish CKD patients from healthy person.

Besides, the cortex R2* values in mild renal damage group and moderate to severe renal damage group have no significantly difference, though had an increasing trend with the decline of renal function. We think it might be limited by the sample size and a larger sample size in future might acquire significantly difference.

Some previous studies showed no significant difference in the medulla R2* values of the BOLD-MRI between the control group and the CKD group (29, 30), which are consistent with our study. On the contrary, other BOLD-MRI studies about medulla R2* values showed that with the decline of GFR or the aggravation of renal damage in CKD patients, the medulla R2* values decreased (12, 24), which suggested that with the decline of renal function, the oxygenation level of the medulla gradually increased. These datas are not consistent with the results of our study. In our study, although the medulla R2* values had a decreasing trend across the three groups, there was no statistical difference. The possible reasons for this are: (1) most of the blood in the kidney is transported to the renal cortex, while only 10% - 15% of blood is sent to the renal medulla (28); as a result, the medulla is relatively not sensitive to hypoxia injury; (2) with the aggravation of renal function damage, the GFR decreases, the ultrafiltration function of the kidney also decreases, and the active absorption of NaCl in the proximal tubules of the medulla weakens; tubular atrophy and reduced active transport of small molecules may lead to reduced Na^+^-K^+^-ATP pump function and reduced oxygen consumption, thereby alleviating renal medulla hypoxia (24); (3) moreover, the R2* values may not only affected by changes in oxygen partial pressure but also by magnetic field strength, homogeneity, pulse parameters, and human physiological data (e.g., pH, temperature, hematocrit) (29) which needs further investigations. Therefore, there was no significant difference in the medulla R2* value among the three groups.

### The cortex FF values in the moderate to severe renal damage group was significantly higher than that in the mild renal damage group

FF values can accurately quantify the lipid deposition in tissue. This study showed that the cortex FF values in the moderate to severe renal damage group was significantly higher than in the mild renal damage group. Previous mDIXON-Quant related studies found that the renal lipid content in the type II diabetes group was significantly higher than that in the non-type II diabetes group, and the corresponding proton-density fat fraction (PDFF) was also higher (20, 31). Another study showed that the renal FF values were higher in patients with early diabetic nephropathy with microalbuminuria compared to those without microalbuminuria and the control group (19). Differently, previous studies measured the renal fat deposition based on the entire renal parenchyma, and we measured the FF values on the cortex and medulla of the kidneys respectively. Besides, previous studies mainly focused on the fat content of diabetic nephropathy, and we expanded the categories of enrolled CKD cases. We also divided the CKD patients into mild renal damage and moderate to severe renal damage group according to the GFR, to explore the feasibility of FF values in the assessment of kidney function in CKD. Similarly, our study and previous studies all indicated that CKD patients have renal fat deposition. The likely reasons may be that obesity and hyperlipidemia are the most common independent risk factors for CKD. A high-fat diet increases the intake of free fatty acids (FFA), CD36 scavenger receptors, fatty acid transporters, and other fatty acids. Overexpression of the uptake system and reduced β-oxidation rate can lead to intracellular lipid accumulation in non-adipose tissue, including kidney. Additionally, excess FFAs can damage podocytes, proximal tubular epithelial cells, and tubular interstitium through multiple mechanisms, especially by promoting the production of reactive oxygen species (ROS) and lipid peroxidation, which in turn promotes mitochondrial damage and tissue inflammation, leading to glomerular and tubular lesions (9, 32). Therefore, CKD renal damage is often accompanied by lipid metabolism disorder and lipid deposition, and the lipid deposition (especially the deposition of FFA) further aggravates renal function damage. Based on those reasons, we speculated that the renal fat deposition increases with the development of the renal injury. Therefore, the cortex FF values of the moderate to severe renal damage group were significantly higher than that of mild renal damage group. Cortex FF values may be helpful to distinguish the degree of renal function damage in CKD patients and stage the diseases.

Yet, in this study, there was no significant difference in medulla FF values among the three groups. And, there was no significant difference in cortex FF values for mild renal damage group vs. healthy control group and moderate to severe renal damage group vs. healthy control group. Theoretically, lipid levels continue to increase with the progression of kidney lesions, but our results does not show this kind of intendency. On the one hand, high fat diet, obesity and body mass index (BMI) may have a certain effect on renal fat deposition, even in healthy people (33). On the other hand, when the renal function is damaged mildly, the kidney has a certain self-regulation mechanism which may help to decrease the lipid content. Besides, there could be local inhomogeneities in fat distribution across different parts of the kidney, and the ROI placements on the renal cortex and medulla may produce some deviation. More research based on this field is needful in future.

The study has a few limitations: (1) this is a retrospective study with a relatively small sample size, which may cause that the range of parameters of one group covers those of other groups. We will expand the sample size to for further study in the future. Also, the patients were not classified according to different etiologies for the high expense of MRI and the emerging technology, and further clinical research on CKD caused by different etiologies is still needed. (2) The kidney structure is heterogeneous, and with the development of renal damage, the boundary between the medulla and cortex was unclear, which may affect the accuracy of ROI placement and measurement; thus, the observer consistency test was performed to reduce measurement errors as much as possible. Besides, we used ROI placements instead of segmentations of the renal cortex and medulla, and there could be local inhomogeneities in fat distribution and deoxygenation across different parts of the kidney; (3) Finally, detailed studies of renal pathology have not been carried out, and our team will improve the research in future.

## Conclusion

The R2* and FF values derived from mDIXON-Quant imaging may reflect the degree of tissue hypoxia and lipid deposition in CKD with different degrees of renal damage. The cortex R2* values have the potential for noninvasively early diagnosis of CKD and earlier intervention. It also can effectively distinguish CKD patients from healthy person and provide useful diagnostic information for physicians. Though the cortex FF values could not be an early indicator for the early diagnosis of CKD, it can be helpful to distinguish the degree of renal function damage in CKD patients and stage the diseases. Therefore, mDIXON-Quant imaging may be an early indicator modality for the non-invasive early diagnosis of CKD, and also can provide a reference for the effective diagnosis, personalized treatment and evaluation of prognosis of CKD.

## Data availability statement

The raw data supporting the conclusions of this article will be made available by the authors, without undue reservation.

## Ethics statement

This study was approved by the Medical Ethics Committee of the First Affiliated Hospital of Dalian Medical University (approval number: PJ-KS-XJS-2022-66). The ethics committee waived the requirement of written informed consent for participation. Written informed consent was obtained from the individual(s) for the publication of any potentially identifiable images or data included in this article.

## Author contributions

Guarantor of the article: AL. Conception and design: AL, YW, and YJ. Collection and assembly of data: YW, YJ, and QA. Data analysis and interpretation: YW and YJ. Manuscript writing: YW. Manuscript editing: YW, AL, YJ, and LL. All authors contributed to the article and approved the submitted version.
