# Corrigendum: mDIXON-Quant for differentiation of renal damage degree in patients with chronic kidney disease

**DOI:** 10.3389/fendo.2023.1267914

**Published:** 2023-08-17

**Authors:** Yue Wang, Ye Ju, Qi An, Liangjie Lin, Ailian Liu

**Affiliations:** ^1^ First Affiliated Hospital, Dalian Medical University, Dalian, China; ^2^ Clinical and Technical Support, Philips Healthcare, Beijing, China

**Keywords:** MRI, mDIXON-Quant, R2*, fat fraction, chronic kidney disease

In the published article, there was an error in the author list, and the co-first authors marks of the first two authors were erroneously excluded. The corrected author list appears below.

Yue Wang^1†^, Ye Ju^1^†, Qi An^1^, Liangjie Lin^2^, Ailian Liu^1*^

(^†^These authors have contributed equally to this work.)

The authors apologize for this error and state that this does not change the scientific conclusions of the article in any way. The original article has been updated.

